# Pharmacy preparedness in handling COVID-19 pandemic: a sharing experience from a Malaysian tertiary hospital

**DOI:** 10.1186/s40545-021-00343-6

**Published:** 2021-07-19

**Authors:** Kah Shuen Thong, Manimegahlai Selvaratanam, Chiew Ping Tan, Meng Fei Cheah, Hoey Lin Oh, Pooi Mun Lee, Chii-Chii Chew, Chee-Tao Chang, Jason Choong Yin Lee

**Affiliations:** 1 Pharmacy Department, Hospital Raja Permaisuri Bainun, Ministry of Health Malaysia, Jalan Raja Ashman Shah, 30450 Ipoh, Perak Malaysia; 2 Clinical Research Centre, Hospital Raja Permaisuri Bainun, Ministry of Health Malaysia, Jalan Raja Ashman Shah, 30450 Ipoh, Perak Malaysia; 3 Perak Pharmaceutical Services Division, Ministry of Health Malaysia, Ipoh, Perak Malaysia

**Keywords:** COVID-19, Pandemics, Pharmacy service, Hospital, Pharmacists

## Abstract

This commentary shares the experience of a hospital pharmacy department in providing healthcare services during the COVID-19 outbreak in Malaysia. During this pandemic, the medication delivery system is redesigned to minimize contact among patients and the health care providers. Also, the remote medication monitoring system was implemented to deliver pharmaceutical care for inpatients. Communication technology was used to assist the pharmacist in medication counseling. QR codes to access videos demonstrating the use of devices were made available for patients. Pharmacists were also tasked with the procurement of personal protective equipment and medications needed requiring special approval from the Ministry of Health.

## Introduction

Malaysian government-funded health facilities are most strongly affected by the COVID-19 (2019 coronavirus disease) pandemic, as 82% of inpatient care is supported by these facilities [1]. One such state-operated tertiary referral hospital is Hospital Raja Permaisuri Bainun, which has a capacity of 990 beds and has been directed to cater to COVID-19 patients from other health facilities within the state of Perak.

The pharmacists in this hospital have been tasked to tackle pharmaceutical care issues and problems during the pandemic. This article is aimed at sharing the experiences of pharmacists in a Malaysian hospital with respect to healthcare preparedness and response during the pandemic. These experiences can inform the formulation of ideas for future research, add to knowledge relevant to hospital pharmacy practice, and serve as reference for health policymakers as they carry out improvements.

### Sustaining supply chains

The lockdown implemented in most countries has caused difficulties for pharmacy departments in procuring medical supplies, particularly medications for the management of COVID-19. Notably, medicines for off-label use as treatment for the disease are not included in the formulary of the Malaysian Ministry of Health (MOH) [2]. The pharmacy department at Hospital Raja Permaisuri Bainun consistently communicates with the pharmaceutical service division at the national level to secure expedited approval to procure the aforementioned medications and supply these to four other specialist hospitals within Perak.

To monitor inventory levels, pharmacists update records of the daily usage of medications on a centralized online database created by the MOH [3]. Hospital pharmacists also constantly communicate with pharmacy departments at the state level to obtain medical supplies for routine use in their hospitals. These responsibilities are in line with the strategies implemented in other countries where pharmacists lead the work involved in the medicine supply chain by conducting active surveillance [4].

To cope with the global shortage in personal protective equipment (PPE) and hand sanitizers [4], multiple strategies have been implemented in this country. These include the creation (sewing) of PPEs by healthcare workers from all disciplines and the acceptance of PPE donations from non-governmental organizations and the public. To ensure the quality of donated PPEs, each item received is inspected by pharmacists and infection control teams. Furthermore, through the national Pharmaceutical Services Programme, a special budget was allocated for the purchase of PPEs and the development of a PPE tracker—an online spreadsheet that all facilities use to update daily PPE balance. To address the temporary shortage of hand sanitizers, certain facilities have produced their own following published formulations [5].

### Redesigning the drug indentation and delivery system

The drug indentation and delivery system in this hospital was redesigned to minimize contact among healthcare providers and patients in the hospital.

### Outpatient pharmacy services

Changes to outpatient pharmacy services were implemented to reduce the number of patients queuing at counters. These changes include dispensing 2 months’ worth of medication supply and extensively promoting pharmacy value-added services (VASs) to the public via direct communication and social media. VASs enable patients to obtain medication refills through their preferred methods, such as delivery by post, drive-through pickup, storage in a designated medicine locker, and acquisition via an integrated drug dispensing system. These services minimize contact between pharmacists and patients and therefore reduce crowding at outpatient pharmacies [6].

To ensure the continuity of care and easy access to medications, the outpatient pharmacy in this hospital continues to operate on a 24-h basis. The patients’ waiting area was modified in accordance with the physical distancing criterion (1-m distance), while pharmacists stationed at the counter are required to wear a face shield over a surgical mask and maintain adequate distance from patients [7].

### Inpatient pharmacy services

We reinforced the medication indentation process through an electronic system called the Pharmacy Information System (PhIS), which enables online prescription, medication supply, medication administration, and counseling documentation. The medicines prescribed by a physician via the PhIS are then prepared by the inpatient pharmacy and distributed to wards. The drug distribution system was modified from a unit-of-dose to a unit-of-use basis, whereby the frequency at which medications are supplied was reduced from daily to 3 times per week. Medication supply to wards involves delivery to a designated work area that is cleaned in accordance with standard protocols. Only medication to be administered is brought to a patient’s bed. Unused medications at the work area are retrieved for decontamination with 70% alcohol solution.

In addition, drug delivery using a medication trolley has been withheld for all COVID-19 wards. All medications are instead packed in a plastic zipper bag for single use [8]. Each bag for each patient contains a 1-week supply of orally administered medications and a 3-day supply of injected forms. Meanwhile, floor stock lists in wards were modified with additional medications required for relieving COVID-19 symptoms; these include chlorpheniramine and diphenhydramine.

### Pharmaceutical care services in wards

In non-COVID-19 wards, to ensure the continuity of core pharmacy services provided to wards, such as medication reconciliation, drug dosage recommendation, and antimicrobial stewardship monitoring, pharmacists wear appropriate PPEs, such as surgical masks and face shields. To practice physical distancing, case discussions among pharmacists and clinicians—commonly held at bedside—are now conducted away from patient cubicles to reduce contact time with patients.

In COVID-19 wards, the entry of healthcare workers is strictly controlled, with full PPE required and only one medical doctor allowed to conduct a clinical assessment of patients. These restrictions are meant to reduce the risk of exposure to the virus among healthcare workers and minimize the consumption of dwindling PPE stocks. Ward pharmacists have taken the initiative to perform remote pharmaceutical care for COVID-19 patients by reviewing drug charts through the PhIS. Interventions are carried out by communicating with prescribers in the wards via telephone call, video conferencing, and messaging through WhatsApp should any pharmaceutical-related problems be detected.

Clinical pharmacists participate in weekly discussions with other healthcare workers using online platforms. Patient cases are summarized, discussed, and given intervention as needed to keep abreast of the latest guidelines and evidence for the use of antibiotics in managing COVID-19. Pharmaceutical care issues related to COVID-19 patients, including drug therapy, dosage, duration of treatment, outcomes, and adverse drug reactions, are reported by clinical pharmacists to the Pharmaceutical Services Programme via a centralized drug monitoring database [3]. These reports serve as an important input for updating COVID-19 management guidelines in Malaysia.

Warded patients requiring assistance with device usage are given pamphlets printed with QR codes that link to a video demonstrating the technique for using a device. Patients are allowed to select their preferred language. An alternative is the recently introduced virtual counseling service, which provides real-time counseling allows pharmacists to interact with patients [9]. However, patients who do not own mobile devices are unable to use these services.

**Fig. 1:**
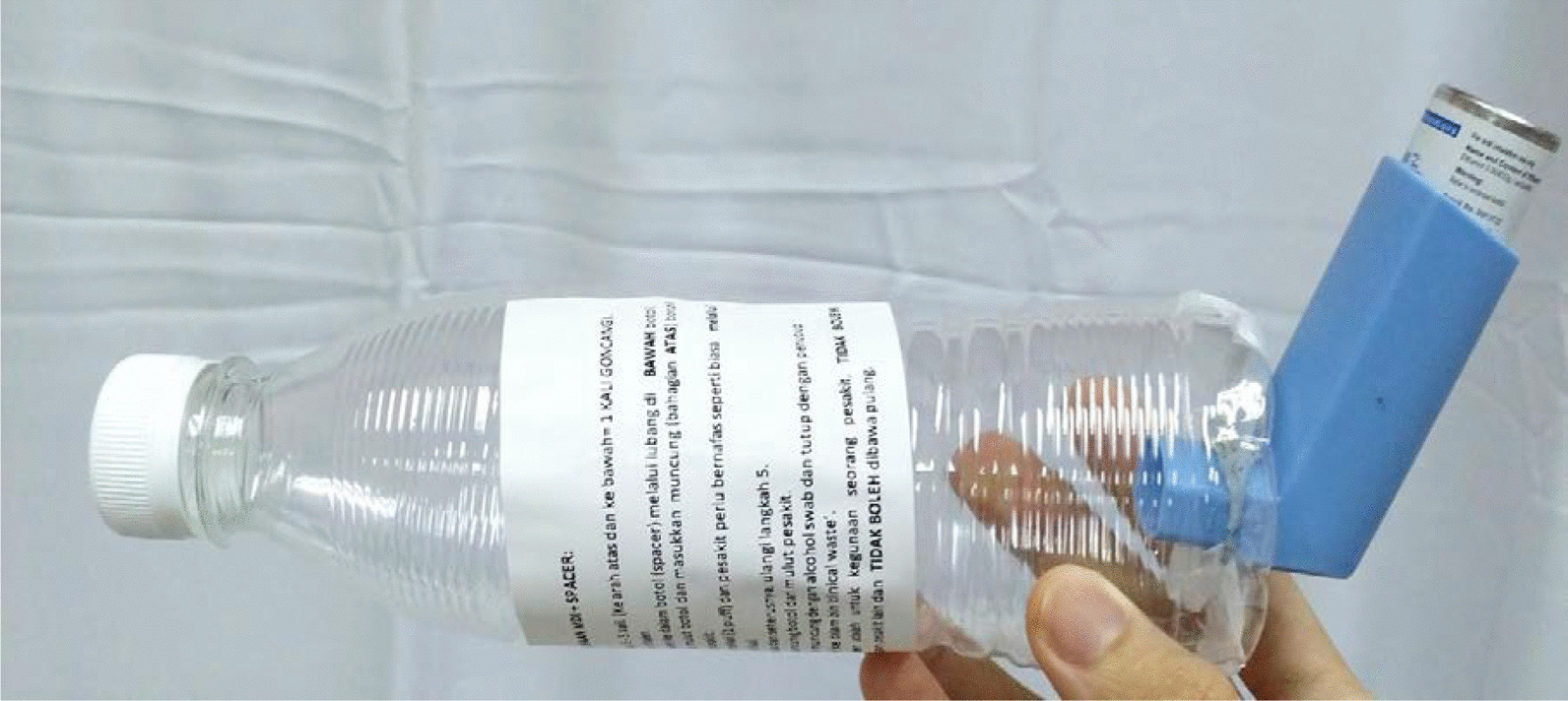
Modified spacer with instruction to use

To minimize aerosol-related transmission, metered dose inhalers are used in place of nebulizers in the treatment of acute asthma in COVID-19 patients. A spacer device is created by modifying a 500-mL plastic bottle, which enables the attachment of a metered dose inhaler to one end of the spacer and the inhalation of medication from the other end (Fig. 1) [10].

## Conclusion

A state hospital’s experience is a valuable learning process that helps pharmacists better prepare for handling future public health emergencies. Limited resources in the field of digital health and a lack of experience in disaster management warrant attention of health policymakers to improve these aspects. Nevertheless, with good leadership, teamwork, and innovative efforts among pharmacists and all healthcare workers, these challenges have been overcome.

## Data Availability

Not applicable.
